# Optimization of a High-Throughput Screen for Monitoring Disease-Associated Protein Misfolding and Aggregation in Bacteria

**DOI:** 10.1021/acssynbio.5c00166

**Published:** 2025-05-12

**Authors:** Dafni C. Delivoria, Eleni Konia, Ilias Matis, Georgios Skretas

**Affiliations:** † Institute of Chemical Biology, 428472 National Hellenic Research Foundation, Athens 11635, Greece; ‡ Institute for Bio-innovation, 54573 Biomedical Sciences Research Center “Alexander Fleming”, Vari 16672, Greece; § Department of Chemistry, University of Crete, Iraklio, Crete 70013, Greece

**Keywords:** protein misfolding diseases, protein aggregation, green fluorescent protein, high-throughput screening, p53, SOD1, amyloid β

## Abstract

Protein misfolding and aggregation are central features of a wide range of diseases, including neurodegenerative disorders, systemic amyloidoses, and cancer. The identification of compounds that can modulate protein folding and aggregation is a key step toward developing effective therapies. High-throughput screening methods are essential for efficiently identifying such compounds. In this study, we optimized a previously developed high-throughput genetic screen for monitoring protein misfolding and aggregation in bacteria. This system is based on monitoring the fluorescence of Escherichia coli cells expressing fusions of human misfolding-prone and disease-related proteins (MisPs) with the green fluorescent protein. We systematically tested a variety of experimental conditions, such as overexpression conditions and MisP-GFP fusion formats, to identify key parameters that affect the sensitivity and dynamic range of the assay. Using misfolding-prone, cancer-associated variants of human p53 as a model system, we found that strong overexpression conditions, such as high copy number vectors, strong promoters, high inducer concentrations, and high overexpression temperatures, can yield optimal assay performance. These optimized assay conditions were also validated with additional MisPs, such as the Alzheimer’s disease-associated amyloid-β peptide and variants of superoxide dismutase 1 associated with amyotrophic lateral sclerosis. At the same time, we observed that certain conditions, such as inducer concentrations and overexpression temperature, may need to be precisely fine-tuned for each new MisP target to yield optimal assay performance. Our findings provide a framework for standardizing MisP-GFP screening assays, facilitating their broad application in the discovery of therapeutic agents targeting protein misfolding and aggregation.

## Graphical abstract

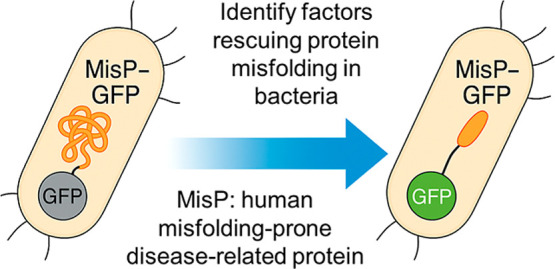

## Introduction

Protein misfolding and aggregation are defining features of numerous human diseases, collectively referred to as protein misfolding diseases (PMDs).^1–5^ These encompass a wide spectrum of disorders, spanning from neurodegenerative conditions like Alzheimer’s disease and Parkinson’s disease to systemic amyloidoses, eye cataracts, type 2 diabetes, and cancer. PMDs impose an enormous socioeconomic impact, and the ongoing lack of effective treatments or disease-modifying therapies, which can prevent, delay, or reverse their progression, necessitates further intensification of research toward their deeper understanding and their treatment through the development of new potent drugs.^1^

Despite their diverse pathologies, a mechanistically unifying feature of PMDs is that they are associated with the misfolding of one or more proteins (misfolding-prone proteins, MisPs). Typically, MisP misfolding is accompanied by a propensity to self-assemble and form aggregates, which can be small or large in size. Smaller aggregates are called oligomers, whereas the larger ones are termed fibrils or, in some cases, amyloids when certain defining criteria are met.^6^ During the last two to three decades, it has been established that either MisP oligomers or fibrils or both are highly toxic for certain cells or tissues in the human body. Thus, conditions that favor the accumulation of misfolded MisP species and of MisP oligomers/fibrils can lead to the development of disease. Consequently, a primary focus in addressing PMDs has been to target the MisP misfolding and aggregation process, either by thermodynamically stabilizing the MisP native state or by kinetically decelerating the MisP aggregation process.^7^ Molecules stabilizing the native MisP are called pharmacological chaperones. Approved anti-PMD drugs with this mechanism of action include the transthyretin stabilizer tafamidis, which is prescribed against familial amyloidosis polyneuropathy and familial/sporadic amyloid cardiomyopathy, as well as migalastat, a small-molecule stabilizer of mutant α-galactosidase A, which is used for the treatment of Fabry disease.^7^ Recently approved drugs functioning as kinetic decelerators of MisP aggregation include the anti-AD monoclonal antibodies lecanemab and donanemab.^7^

Once a MisP drug target has been identified, the next step in the drug discovery pipeline involves the development and implementation of screening assays to identify hits and, subsequently, promising therapeutic leads. Indeed, an extensive number of screening assays have been developed against PMDs, which can be divided into three main categories: computational, in vitro biochemical/biophysical, and phenotypic.^8–18^ Among these, the latter presents a valuable opportunity for investigating MisP misfolding and aggregation in the physiologically relevant environment of a living cell or organism. Notably, various aspects of disease-related protein misfolding and aggregation can be faithfully replicated in microorganisms of both prokaryotic and eukaryotic cells, and, thus, such simple hosts can be utilized for the development of high-throughput and cost-effective screening methods that facilitate the identification of effective hits.^18,19^ The use of Escherichia coli offers several advantages as a microbial host—such as ease of genetic manipulation, rapid growth, cost-effective cultivation, and maximal transformation efficiencies allowing screening of very large biomolecular libraries. At the same time, E. coli can also present certain limitations for studying human protein misfolding. One major drawback is the absence of post-translational modifications typically present in eukaryotic cells, particularly protein glycosylation. Many human proteins undergo N- or O-linked glycosylation, which can influence their folding, stability, and aggregation propensity. As such, bacterial systems may not fully recapitulate the native folding or misfolding behavior of glycosylated proteins, potentially affecting the physiological relevance of the screening results. Additionally, differences in translation rates, molecular chaperone systems, and proteostasis networks between E. coli and human cells may impact protein folding outcomes. Eukaryotic microbial systems, most notably yeast species, such as Saccharomyces cerevisiae, have also been employed in protein misfolding studies. These organisms are capable of performing some types of post-translational modifications in a human-like manner and offer a more eukaryote-like intracellular environment, making them valuable complementary platforms for confirming and expanding findings from bacterial screens.

A commonly utilized approach for identifying rescuers of MisP misfolding and aggregation in microbial hosts involves the use of genetic systems, where the folding/aggregation status of the MisP of interest is coupled with that of a reporter protein (RP). In these systems, genetic fusions of the target MisP with the selected RP are first generated. These fusions can be either end-to-end (RP attached to the N or C terminus of the target MisP) or insertional, where the MisP is integrated into an internal region of the RP.^18,20–23^ The MisP and the RP are appropriately connected with one another in a way so that the (mis)­folding status of the MisP is communicated to the RP, thus resulting in variable levels of reporter activity and consequently to phenotypic changes of the cell host producing the MisP-RP fusion, such as growth, color, or fluorescence.

Among these, end-to-end MisP fusion pairs with fluorescent proteins, such as the green fluorescent protein (GFP), have been extensively employed due to the ability for facile monitoring of RP activity either by the naked eye or by using standard laboratory equipment, such as plate readers and flow cytometers. In this genetic screen, GFP is attached to the C terminus of the target MisP and is expressed in a microbial host, such as E. coli. Due to the tendency of the target MisP for misfolding and/or aggregation, MisP-GFP overexpression leads to the production of similarly misfolded fusion proteins and to their subsequent accumulation in insoluble inclusion bodies exhibiting little or no fluorescence. Thus, the bacterial cells expressing such MisP-GFP fusions under normal circumstances exhibit low fluorescence levels (Figure 1). Conversely, conditions that rescue protein misfolding and/or inhibit aggregation result in the production of better folded, soluble, and fluorescent MisP-GFP chimeras, and the bacterial cells producing these fusions under such conditions exhibit increased fluorescence and can be easily detected.

**Figure 1:**
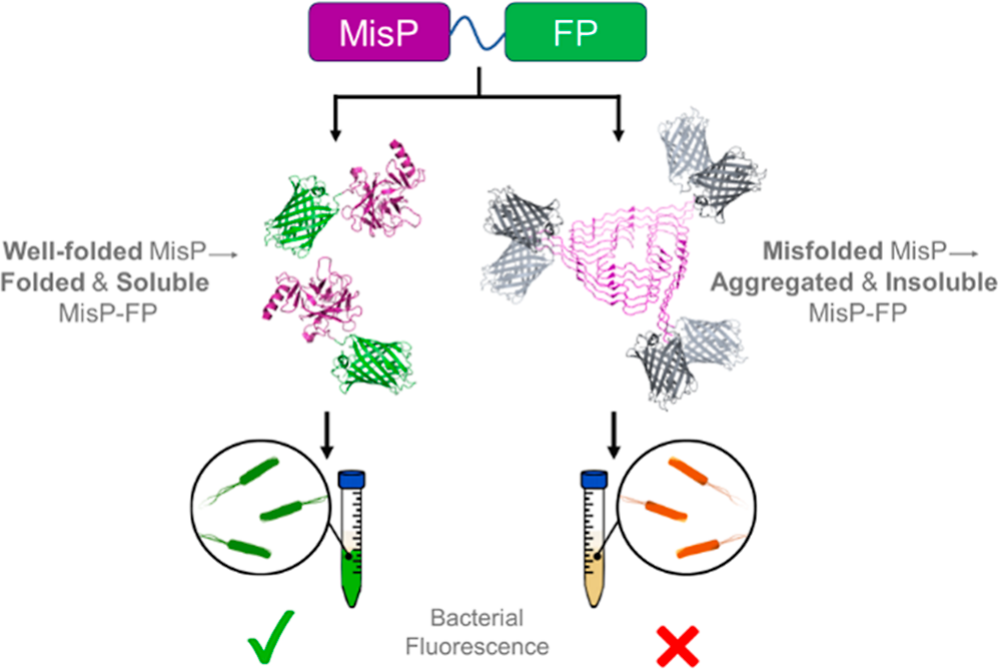
Schematic of the MisP-FP genetic system for monitoring MisP folding and aggregation. Due to the tendency of MisPs to misfold and aggregate, overexpression of a MisP-FP chimera in E. coli results in the accumulation of the protein in insoluble inclusion bodies that lack fluorescence. Thus, bacterial cells expressing MisP-FP fusions exhibit low fluorescence. On the contrary, in the presence of factors that rescue MisP misfolding and/or inhibit MisP aggregation, MisP-FP is produced in soluble and fluorescent form, and thus, E. coli cells expressing this fusion under these conditions exhibit increased fluorescence.

This approach was initially inspired by the work of Waldo et al., who utilized this system as a facile way for monitoring the levels of soluble recombinant proteins, which can be produced in bacteria.^24^ Later on, its use was extended to target disease-related MisPs by Hecht and co-workers, as well as by Ventura and co-workers, who created chimeras of the AD-associated amyloid-β peptide 1–42 (Aβ42) with GFP to identify factors, such as amino acid substitutions in the sequence of Αβ42, that affect the aggregation of Aβ42.^25,26^ Subsequently, various research groups have utilized this assay as a screening tool to identify small-molecule or peptide inhibitors of Aβ42 aggregation.^27–29^ Notably, the MisP-GFP screen has also been applied to study the folding, aggregation, and/or stability of other disease-associated MisPs, such as the islet amyloid polypeptide (IAPP), which is related to type 2 diabetes^30^ and p53 variants related to carcinogenesis.^31^ More recently, we have exploited the capabilities of this assay to develop an integrated bacterial system allowing the biosynthesis of combinatorial libraries of hundreds of millions of short, drug-like cyclic peptides and their simultaneous functional screening for identifying rescuers of pathogenic protein misfolding and aggregation using ultrahigh-throughput flow cytometric sorting.^32–35^ We applied this system against Aβ42 and the A4V variant of the human Cu/Zn superoxide dismutase (SOD1­(A4V)), which is associated with a familial form of ALS, and have identified hundreds of hits, which were found to function as inhibitors of pathogenic protein aggregation both in vitro and in vivo.^32,33^

In the present work, we systematically examined the factors and conditions that influence the efficiency of the MisP-GFP system to optimize the assay performance and facilitate future screening efforts employing this system. We demonstrate that, in general, conditions known to drive protein misfolding, such as strong overexpression (e.g., use of high copy number vectors, strong promoters, high inducer concentrations, etc.) and high overexpression temperatures, can yield optimal assay performance. In addition, we observed that certain optimized conditions were broadly applicable to different MisP targets, while others varied depending on the specific MisP being studied, thus highlighting the need for further optimization for each new target in these cases. Our present work provides experimental guidelines that should allow the MisP-GFP system to find widespread application as a high-throughput screening tool in the search for agents capable of rescuing pathogenic protein misfolding.

## Results and Discussion

A relevant literature search revealed that the published screens utilizing the MisP-GFP system were performed by using a range of different overexpression conditions for MisP-GFP fusion production. Based on this, we opted to examine whether these can have a significant effect on assay performance and whether we can determine more widely applicable optimal overexpression conditions for this assay. In this scope and as an initial example of a MisP, we selected the human tumor suppressor protein p53. p53 is a transcription factor, which plays a crucial role in protecting cells against uncontrolled proliferation and carcinogenesis and is often referred to as the “guardian of the genome”.^36^ The significance of the protective function of p53 is underscored by its widespread inactivation in a multitude of cancers, either through mutations in the *TP53* gene or deregulations of its signaling pathways.^37^ The majority of *TP53* mutations occur within the DNA-binding (core) domain of the protein (p53C) and can be categorized into two types: (i) contact mutations, which occur within or near the DNA-binding domain of p53, directly hindering p53-DNA binding, and (ii) structural mutations, which are located at the periphery of the core domain, inducing changes in protein conformation and/or stability.^38^ Due to the marginal stability of p53, these changes can lead to protein misfolding and consequent inactivation at normal body temperature.^38^ We selected p53C as our initial target MisP because: (i) Fersht and co-workers have employed the MisP-GFP system to illustrate that the thermodynamic stability of diverse p53C structural mutants correlates well with the fluorescence and aggregation propensity of their GFP fusions when overexpressed in E. coli,^31^ and (ii) structural p53 variants constitute major targets for drug discovery.^38–41^ Interestingly, misfolding rescuers for structural p53 variants have recently entered clinical testing.^42^

We selected to include in our study three disease-associated structural mutations into p53C, namely the substitution of valine at position 143 by alanine (V143A), the substitution of tyrosine at position 220 by cysteine (Y220C), and the substitution of phenylalanine at position 270 by leucine (F270L). These substitutions are located at different regions of the tertiary structure of p53, they have been found to have variable effects on p53 stability and folding, and they have all been found in tumors.^39,43^ Also, some of these variants have been the target of candidate drugs currently in clinical development.^42^ These mutations were introduced into the sequences of both p53C and the highly stabilized p53 variant T-p53C. T-p53C harbors four point mutations (M133L, V203A, N239Y, and N268D) that increase thermal stability, reduce aggregation, and simplify experimental handling while maintaining an almost identical structure to the wild-type protein.^39,44,45^ We generated recombinant fusions of the aforementioned p53 variants with GFP+, a GFP variant containing the substitutions F64L, S65T, Q80R, F99S, M153T, and V163A,^46^ comprising the more soluble and fluorescent variant contained in the original vector constructed by Waldo et al.^24^ and overexpressed them under the control of the strong T7 promoter in E. coli. Then, we monitored the bacterial fluorescence and tendency for aggregation by monitoring the accumulation of the p53-GFP protein in the soluble versus insoluble fractions. As also previously observed for other p53 variants,^47^ we confirmed that the fluorescence intensity of E. coli cells expressing p53C-GFP fusions correlated very well with their thermodynamic stability and the accumulating amounts of soluble protein (Figure 2, Supplementary Figure 1).

**Figure 2:**
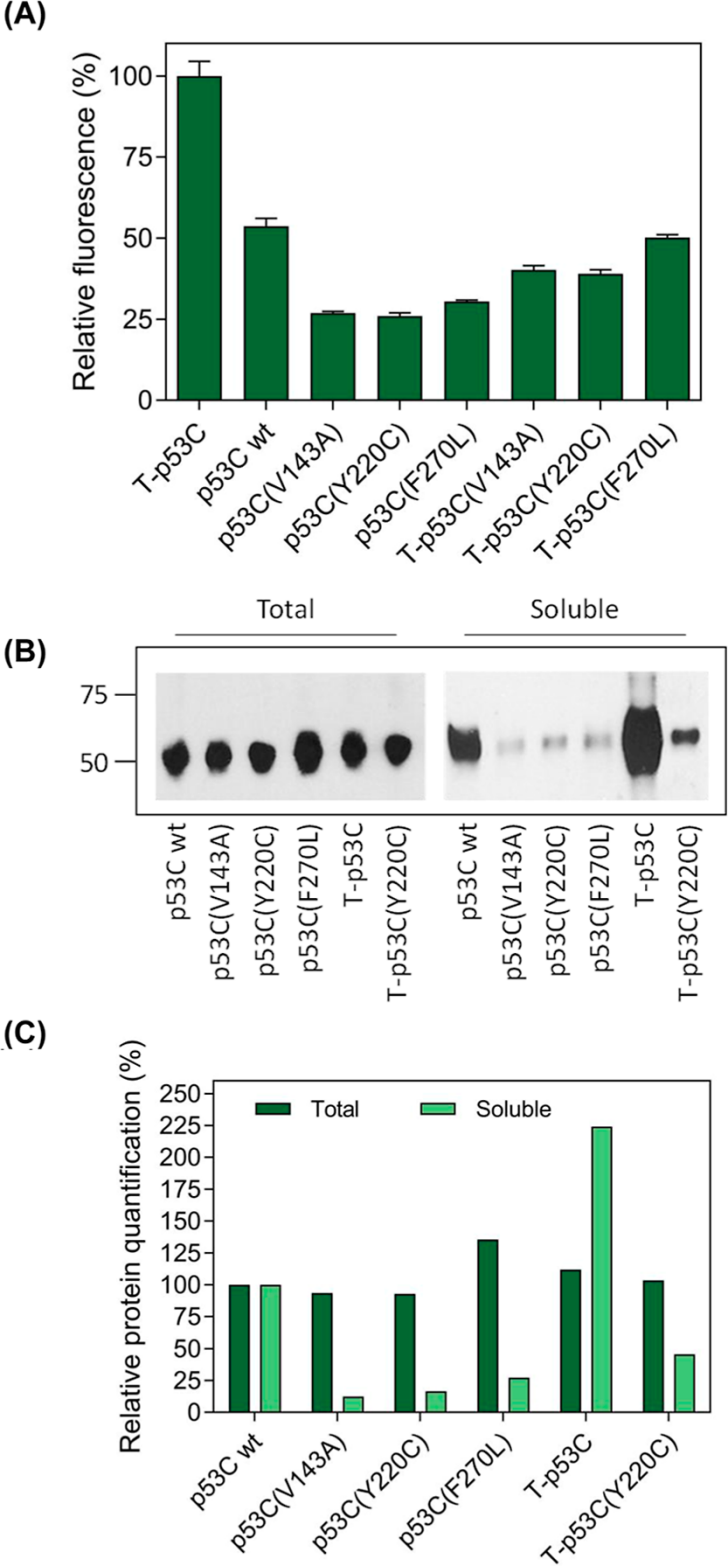
Monitoring the misfolding and aggregation of oncogenic p53 variants using the MisP-GFP assay. (a) Relative fluorescence of E. coli BL21 (DE3) cells overexpressing p53C-GFP fusions from pET28-p53-GFP vectors for 2 h at 37 °C and using 0.1 mM IPTG. Relative mean values ±s.e.m. of experiments performed in triplicates are reported. Bacterial fluorescence was measured using a plate reader, and the fluorescence of the bacterial population producing T-p53-GFP was arbitrarily set to 100. (b) Solubility analysis of E. coli BL21 (DE3) cells overexpressing p53C-GFP fusions. Total (left) and soluble (right) lysates of cells overexpressing different p53C-GFP fusions produced as in (a) were analyzed by SDS-PAGE and visualized by Western blotting using an anti-GFP antibody. The molecular mass of the DNA-binding domain of human p53 (core domain; p53C) is ∼25 kDa. (c) Densitometric quantification of Western blot bands shown in (b). Band intensities corresponding to total and soluble fractions were quantified using ImageJ. The intensities corresponding to the total and soluble fractions of p53C wt were arbitrarily set to 100 for normalization.

To initiate the optimization procedure, we chose as a proxy for the efficiency of the assay the difference in fluorescence between p53-GFP fusions containing a more stable, better folded, and less aggregation-prone p53 variant, such as T-p53C, and a less stable and misfolding/aggregation-prone variant, such as p53C­(Y220C). We started by testing a variety of different expression vectors containing a range of different promoters and origins of replication (Supporting Information Table S1). The selected plasmids comprised a selection of promoters with varying strengths, such as the very strong *P*_T7lac_ and *P*_tet_, the robust *P*_trc_ and the moderately strong *P*_BAD_, as well as a selection of origins of replication (ori), such as the pBR322 ori with >40 plasmid copies per cell and the p15A ori with ∼10 copies per cell.^48–50^ We found that strong overexpression conditions like the ones occurring by the use of stronger promoters from high copy number vectors, such as the ones in the expression vectors pET28 (pBR322 ori, T7 promoter) and pASK75 (pBR322 ori, Tet promoter), resulted in increased differences in fluorescence intensities between the more stable, better folded, and less aggregation-prone T-p53C compared to the less stable and more aggregation-prone variant T-p53C­(Y220C), thus indicating that these conditions provide a larger dynamic range and are, thus, better suited for monitoring MisP misfolding and aggregation using this assay (Figure 3a).

**Figure 3:**
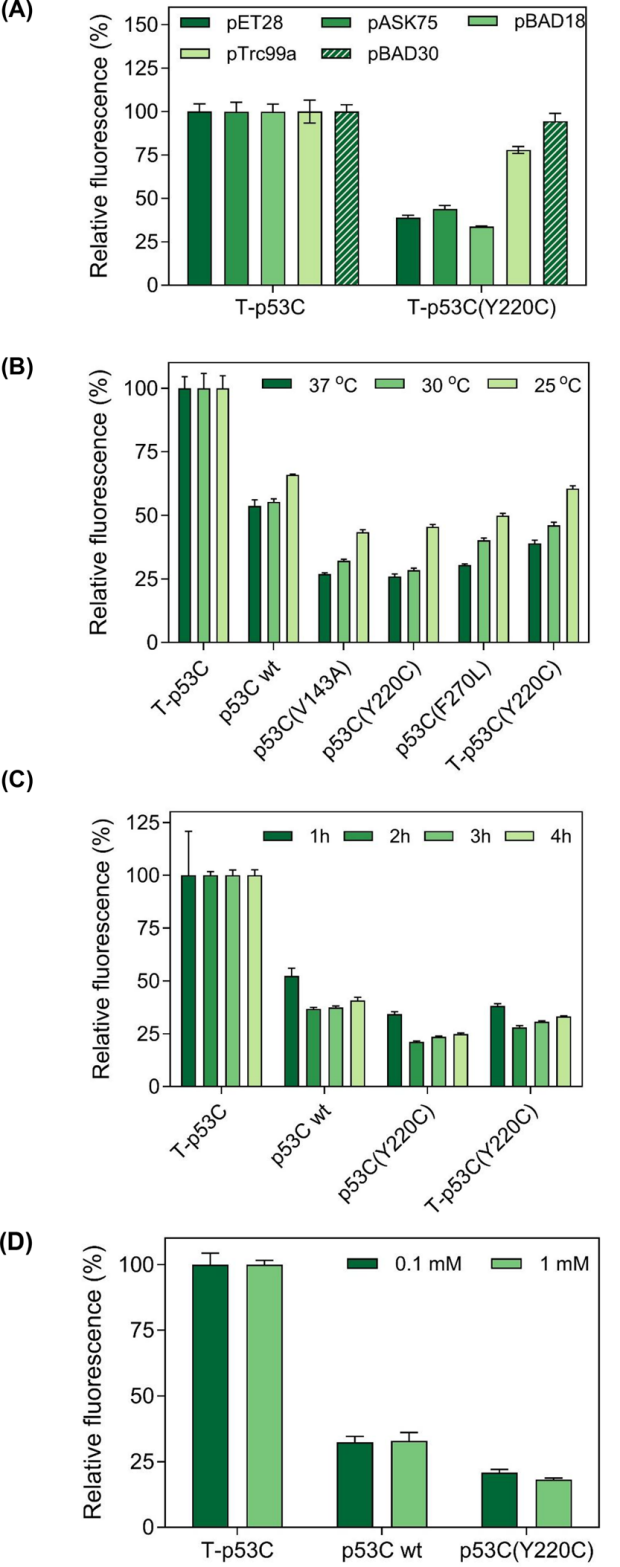
Effect of different optimization parameters on the bacterial fluorescence of E. coli BL21­(DE3) cells producing p53C-GFP fusions. (a) Investigation of different expression vectors, (b) incubation temperatures, (c) incubation periods, and (d) IPTG concentrations. In all panels, overexpression was performed using the pET28 vector, unless otherwise stated, and the fluorescence of the bacterial population producing T-p53C was arbitrarily set to 100. In (a), protein overexpression was induced by the addition of 0.1 mM IPTG for pET28 and pTrc99a, 0.2 μg/mL anhydrotetracycline (aTc) for pASK75, and 0.02% l­(+)-arabinose for pBAD18 and pBAD30 vectors for 2 h at 37 °C in all cases. In (b), induction was performed with 0.1 mM IPTG, and cells were incubated postinduction for 2 h at 37 °C, 5 h at 30 °C, or 16 h at 25 °C. In (c) overexpression was performed using 0.1 mM IPTG in all cases. In (d), induction was carried out for 2 h at 37 °C for both tested IPTG concentrations. Mean values ±s.e.m. are presented in all cases. Each experiment was performed in triplicates. In all panels, bacterial fluorescence was measured using a plate reader.

Next, we assessed the impact of varying the incubation temperatures and time periods on the production of the p53C-GFP fusions. We observed that the differences in fluorescence intensity between T-p53C and T-p53C­(Y220C) decreased with decreasing temperature (Figure 3b). This outcome was in line with our expectations, as it is well-established that p53­(Y220C) folding can be restored to near wt levels at temperatures below 37 °C.^38^ Full restoration of the p53C­(Y220C) misfolding effect could not be observed here due to (i) the high overexpression effect achieved with the strong T7 promoter, which exacerbates misfolding propensity, and (ii) because overexpression temperatures below 25 °C were not tested, which are probably required to achieve this. Regarding the duration of the overexpression process, we found that 2–3 h of induction of protein overexpression upon addition of isopropyl β-D-thiogalactoside (IPTG) are optimal for maximizing the differences between T-p53C-GFP and T-p53C­(Y220C)-GFP fluorescence (Figure 3c).

In parallel, we examined how varying the concentration of the inducer affected the assay performance. Interestingly, we found that elevating the inducer concentration from 0.1 mM to 1 mM had a marginal effect on the differences in fluorescence intensity between T-p53C and T-p53C­(Y220C) (Figure 3d). Furthermore, the higher inducer concentrations (1 mM) resulted in considerable cellular toxicity as manifested by the ∼30% levels of final biomass of the bacterial cultures and in accordance with the literature.^51^ Thus, intermediate inducer concentrations offering a maximal dynamic range, while also avoiding substantial stress and allowing healthy growth, appear to be optimal for the MisP-GFP screen.

Having established optimal overexpression conditions for p53C-GFP fusions, we tested whether the originally utilized GFP+ is the ideal fluorescence partner for monitoring protein misfolding and aggregation in a MisP-RP fusion setup. For this, we produced recombinant fusions of p53C variants with other fluorescent proteins, such as the blue fluorescent protein (BFP)^52^ and the red fluorescent protein (RFP),^53^ and measured the fluorescence of E. coli cells expressing these fusions. T-p53C and T-p53C­(Y220C) fusions with GFP+ and BFP exhibited similar differences in fluorescence intensity, thus indicating that both fluorescent proteins can be employed for monitoring protein misfolding and aggregation equally efficiently (Figure 4a). On the contrary, the differences in T-p53C-RFP and T-p53C­(Y220C)-RFP fluorescence intensities were decreased compared to the GFP+ and BFP fusions, indicating that this fluorescent partner may be less efficient for this purpose (Figure 4a).

**Figure 4:**
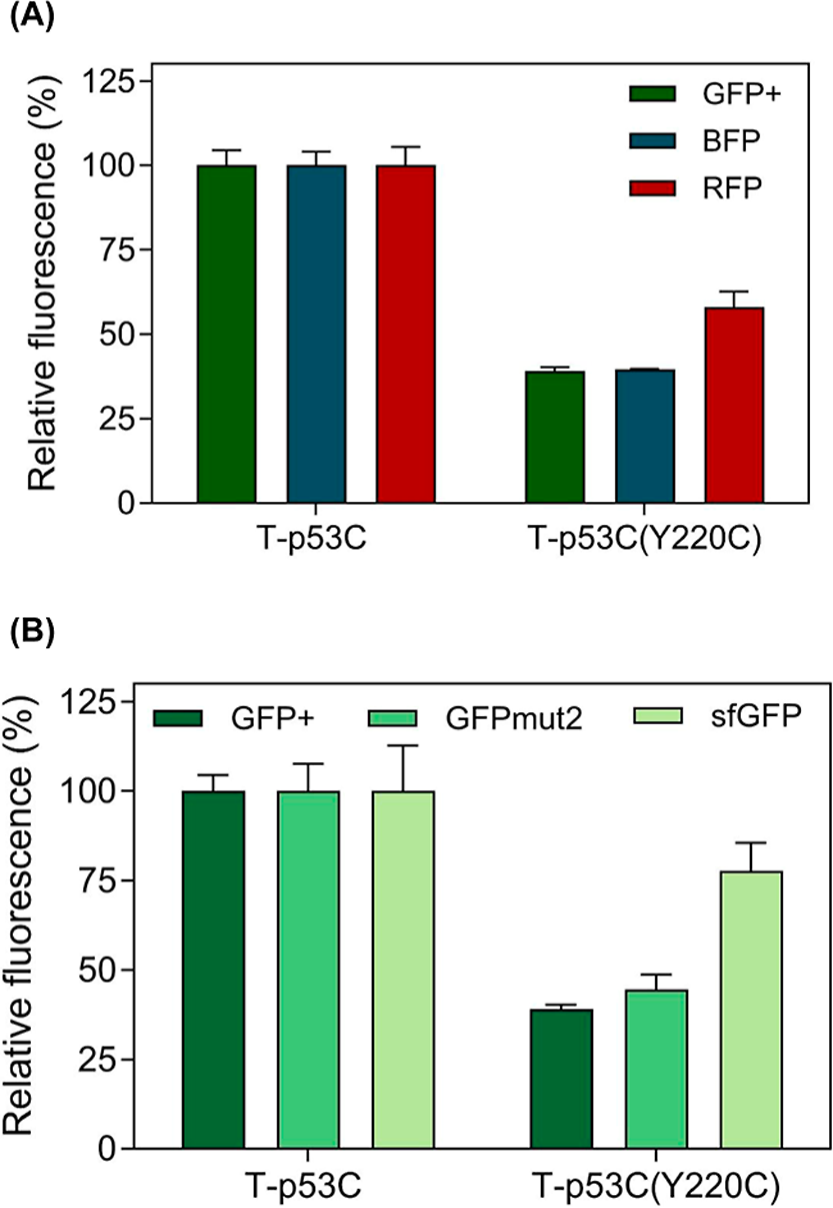
Evaluation of different fluorescent protein partners for monitoring protein misfolding and aggregation. (a) Effect of different fluorescent protein partners (green, blue, or red fluorescent proteins) for monitoring protein aggregation in E. coli Tuner­(DE3) cells. Protein production was performed for 2 h at 37 °C using the pET28 vector and 0.1 mM IPTG, and the bacterial population producing T-p53C was arbitrarily set to 100. Mean values ±s.e.m. are presented. Each experiment was performed in triplicates. (b) Comparison of GFP variants as fusion proteins for monitoring protein aggregation in E. coli. Protein production was performed for 2 h at 37 °C, using the pET28 vector and 0.1 mM IPTG. The bacterial population producing T-p53C was arbitrarily set to 100. Mean values ±s.e.m. are presented. In both panels, bacterial fluorescence was measured by using a plate reader. Tuner host strains are *lacZY* deletion mutants of E. coli BL21­(DE3) enabling more adjustable levels of protein expression more uniformly throughout all cells in a culture.

Furthermore, we tested for possible differences in performance among different frequently utilized variants of the GFP reporter, GFPmut2 (containing the substitutions S65A, V68L, and S72A)^54^ and superfolder GFP (sfGFP; containing the substitutions S2R, S30R, Y39N, F64L, S65T, Q80R, F99S, N105T, Y145F, M153T, V163A, I171V, and A206V).^55^ We found that the activity of all GFP variants was affected by the (mis)­folding of their upstream fusion protein partner, as indicated by the fluorescence levels of the T-p53C and T-p53C­(Y220C) fusions when fused with these RPs. The more stable sfGFP, however, showed greater resilience, exhibiting only slight decreases in fluorescence when paired with the more misfolding-prone Y220C variant (Figure 4b).

Next, we aimed to investigate whether the optimal arrangement involved fusing the MisP of interest with GFP+ in an end-to-end or insertional manner. We reasoned that MisP domain insertion into GFP may cause a greater interdependence of the fluorescence levels of the reporter on the folding status of the inserted MisP, as has been observed for other similar genetic screens and selections for protein folding.^20^ To investigate this hypothesis, we utilized two sites, which have been previously found to be permissive for domain insertions in GFP: one at loop 8 between Gln157 and Lys158, and one at loop 9 between Glu172 and Asp173.^56^ Interestingly, contrary to our expectation, coupling the MisP of interest to GFP+ in an end-to-end manner proved to be significantly more effective for monitoring the tendency for MisP misfolding and aggregation (Figure 5).

**Figure 5:**
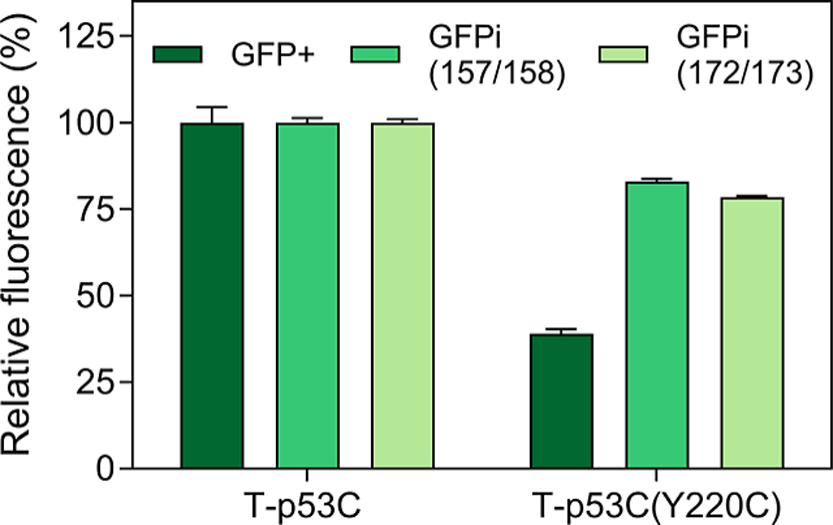
Evaluation of different fusion strategies for monitoring protein misfolding and aggregation. Protein production was performed in E. coli Tuner­(DE3) for 2 h at 37 °C using the pET28 vector and 0.1 mM IPTG and the bacterial population producing T-p53C was arbitrarily set to 100.

Subsequently, we reasoned that the interaction between the MisP of interest with GFP and the coupling of the (mis) folding status of the MisP target to the activity of the fluorescent fusion partner could be impacted by the properties of the linker connecting these two distinct domains. To address this, we tested a selection of linkers of diverse nature (i.e., flexible or rigid), length (ranging from 0 to 16 aa), and codon frequency (i.e., rare or frequent) to identify the most appropriate linker ((Supporting Information Table S2). Specifically, apart from employing the linker with the sequence GSAGSAAGSGEF, as originally selected by Waldo et al.,^24^ which was extended by two amino acids (LQ; linker Waldo­(ext)) to facilitate cloning and applied in the aforementioned experiments, we also tested the p53C-GFP fusions containing the following linkers: (i) seamless fusions without the use of any linker; (ii) a (Gly_4_Ser)_2_ flexible linker, where Gly and Ser are encoded by the more frequently utilized codons GGC/GGT and TCC/TCG in E. coli, referred to as (Gly_4_/Ser)_2_^F^; (ii) a (Gly_4_/Ser)_2_ flexible linker, where Gly and Ser are encoded by the less frequently utilized codons GGG/GGA and TCΑ/TCC in E. coli, referred to as (Gly_4_/Ser)_2_^R^); and (iv) the rigid linker (EAAAK)_3_. As shown in Figure 6, the use of different linkers yielded similar differences in fluorescence between T-p53C-GFP and T-p53C­(Y220C)-GFP fluorescence, thus indicating that linker selection is not a critical factor for assay performance in the MisP-GFP screen. This is consistent with the original observations by Waldo et al., who have reported that a longer (GGGS)_3_ linker was also tried but did not appear to change the performance of the MisP-GFP folding reporter.^24^

**Figure 6:**
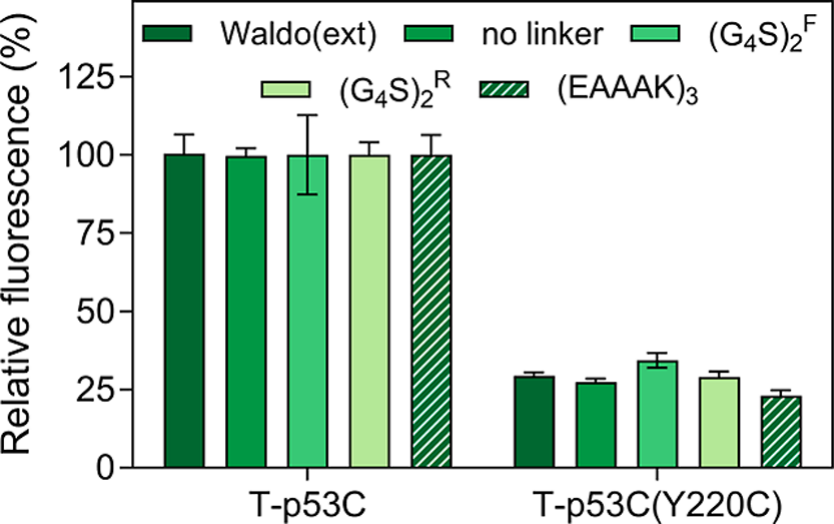
Impact of linker properties on the interaction between MisP and GFP and the detection of protein misfolding in E. coli. Protein production was performed in E. coli Tuner­(DE3) cells for 2 h at 37 °C using the pET28 vector and 0.1 mM IPTG. For each linker, the bacterial population producing T-p53C was arbitrarily set to 100. Mean values ±s.e.m. are presented. Each experiment was performed in triplicates. Bacterial fluorescence was measured using a plate reader.

After determining the optimal conditions for monitoring the misfolding and aggregation using the GFP genetic screen using p53 as a test case, we aimed to evaluate whether these are also extendable and applicable to other MisP proteins as well. In this scope, we opted for two unrelated MisPs: human SOD1 and Aβ42. Regarding Aβ, an intrinsically disordered polypeptide, we compared two variants, the highly aggregation-prone Aβ42 and the engineered variant Αβ42­(F19S; L34P), exhibiting significantly decreased aggregation propensity and enhanced solubility.^25^ For SOD1, a globular dimeric enzyme, we compared the highly stable wild-type protein (SOD1wt) with four ALS-linked variants exhibiting enhanced misfolding and aggregation propensities, namely the substitution of alanine at position 4 by valine (A4V), the substitution of glycine at position 37 by arginine (G37R), the substitution of glycine at position 85 by arginine (G85R), and the substitution of glycine at position 93 by alanine (G93R).^57^ We proceeded to test variations of MisP-GFP overexpression conditions, such as inducer concentrations and incubation temperatures, and evaluated whether strong overexpression can enable efficient monitoring of MisP misfolding and aggregation for Aβ and SOD1 as in the case of p53. Indeed, Aβ42- and SOD1-GFP overexpression from high copy number vectors, such as pET28, at higher temperatures of 30–37 °C and with medium to high inducer concentrations (0.1–1 mM IPTG for Αβ and 0.01–0.1 mM IPTG for SOD1), yielded very clear fluorescence differences between the more and less misfolding/aggregation-prone variants tested from both MisPs (Figures 7 and 8). These differences in fluorescence could be observed at similar levels when bacterial fluorescence was measured using a plate reader (Figures 7 and 8) or flow cytometry, in which case cultures with good fluorescence homogeneity could be observed (Supporting Information Figure S2). Importantly, the fluorescence of bacterial cells expressing these MisP-GFP fusions correlated well with the amount of protein accumulating in the soluble cellular fraction and was inversely proportional to inclusion body formation (Figures 7c and 8b).

**Figure 7:**
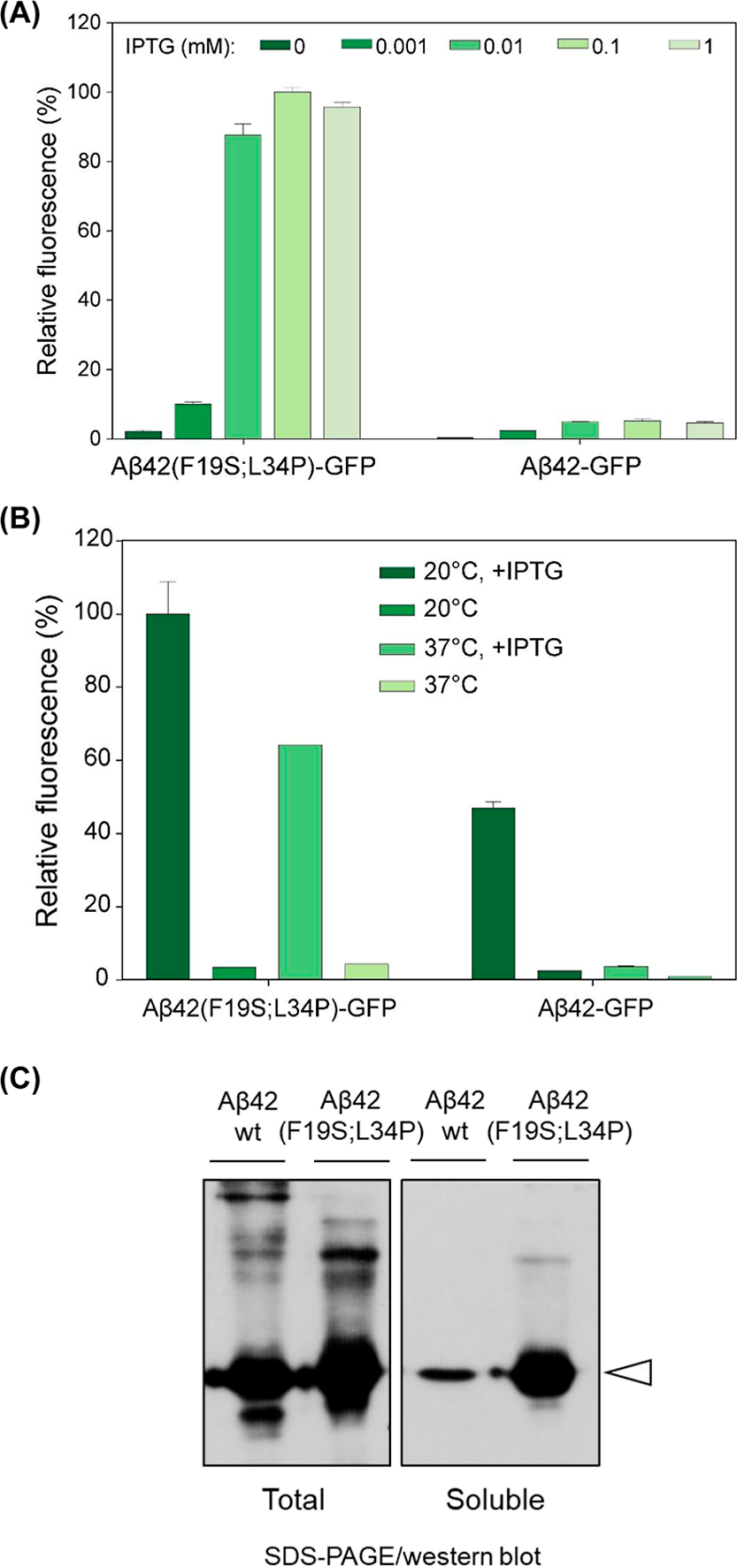
Application of the MisP-GFP genetic screen to monitor misfolding and aggregation for other MisPs—the case of Αβ. (a) Comparison of the fluorescence of E. coli BL21­(DE3) cells overexpressing Αβ42-GFP fusions containing wild-type (wt) Αβ42 and the less aggregation-prone variant Αβ42­(F19S; L34P) using the pET28 vector in the presence of varying concentrations of the inducer IPTG for 2 h at 37 °C. (b) Effect of varying incubation temperatures on the bacterial fluorescence of E. coli BL21­(DE3) cells overexpressing Αβ42-GFP as in (a) in the presence of 0.1 mM IPTG for 2 h at the indicated temperatures. Mean values ±s.e.m. are presented. Each experiment was performed in triplicates. In (a,b), bacterial fluorescence was measured using a plate reader. (c) SDS-PAGE/Western blot analysis of total (left) and soluble (right) lysates of E. coli BL21­(DE3) cells coexpressing Aβ42-GFP and Αβ42­(F19S; L34P)-GFP for the pET-Αβ-GFP vector in the presence of 0.1 mM IPTG at 37 °C for 2 h, probed with an anti-GFP antibody. The Aβ42-GFP fusion is indicated by the arrow.

**Figure 8:**
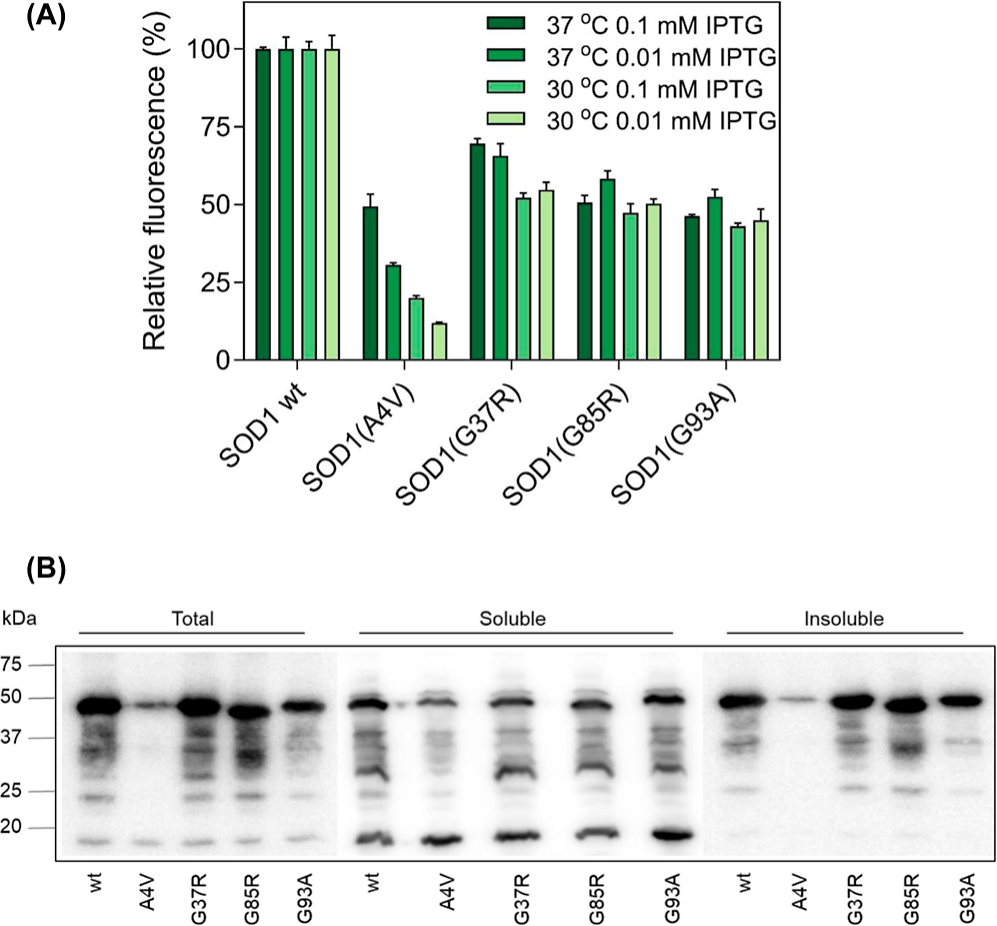
Application of the MisP-GFP genetic screen to monitor misfolding and aggregation for other MisPs—the case of SOD1. (a) Comparison of fluorescence of E. coli Origami2­(DE3) cells expressing SOD1-GFP fusions containing wild-type (wt) SOD1, SOD1­(A4 V), SOD1­(G37R), SOD1­(G85R), and SOD1­(G93A) from the pET28 vector at different incubation temperatures and IPTG concentrations. The fluorescence of the bacterial population producing SOD1wt was arbitrarily set to 100. Mean values ±s.e.m. are presented. Each experiment was performed in triplicates, and bacterial fluorescence was measured using a plate reader. (b) Solubility analysis by SDS-PAGE/Western blotting using an anti-polyHis antibody of SOD1-GFP fusions overexpressed in E. coli Origami 2­(DE3) cells from the corresponding pETSOD1-GFP vectors following the addition of 0.01 mM IPTG at 37 °C for 2 h.

Interestingly, we found that for SOD1, and compared to p53 and Αβ, somewhat milder overexpression conditions, i.e., 0.01–0.1 mM IPTG at 30 °C, resulted in maximal differences in the fluorescence intensity of wild-type SOD1 and the disease-associated variants, most notably for the most aggregation-prone variant A4V (Figure 8a). Indeed, in our previous screens to identify cyclic oligopeptide rescuers of SOD1­(A4V) misfolding and aggregation using the MisP-GFP assay, lower IPTG concentrations were utilized for SOD1 compared to the Αβ screen, as SOD1­(A4V) overexpression under these milder overexpression conditions proved to be much less toxic for bacteria and allowed for more efficient screening.^33^ These results indicate that although some general guidelines can be provided for factors and conditions that yield good performance in the MisP-GFP screen, there are also some expression parameters, such as inducer concentration and overexpression temperature, which may have to be fine-tuned whenever a new target MisP is selected. It seems likely that more challenging or more toxic for the cell host MisP targets may require milder overexpression conditions, whereas less problematic MisPs would yield maximal MisP-GFP signal differences under conditions that challenge the folding of the MisP target to a larger extent.

In conclusion, our present study has explored systematically how different factors can affect the assay performance in a high-throughput genetic screen employing bacteria expressing MisP-GFP fusions and recording cell fluorescence to monitor disease-related protein misfolding and aggregation. We have identified parameters that consistently yield a high assay performance in the MisP-GFP screen for a variety of different MisP targets. These include the use of high-copy number vectors, strong promoters, medium to high inducer concentrations, and higher overexpression temperatures (30–37 °C). At the same time, the exact inducer concentrations and overexpression temperatures yielding maximal signal differences between better and worse folded MisPs may require more precise fine-tuning every time a new MisP target is considered. Furthermore, we have found that different fluorescent proteins, such as GFP+, GFPmut2, and BFP, can be used as RPs equally efficiently in this assay. Interestingly, we have observed that MisP misfolding and aggregation propensity can be sensed by the reporter fluorescent protein significantly more effectively when the two domains are connected as part of an end-to-end format compared to an insertional fusion format. On the contrary, the length or nature of the amino acid linker that connects MisP and the GFP domains does not appear to have a significant impact on the performance of the assay. Overall, using the guidelines described herein, one can select conditions that yield good performance in the MisP-GFP screen. This system can serve as a valuable tool for studying certain molecular mechanisms underlying pathogenic protein misfolding in a simple living organism, which, despite the fact that the intracellular space of a microorganism is in many ways very different from that of a human cell, can offer a much more physiologically relevant environment for this purpose than that of a test tube containing isolated protein in buffer.^58^ Furthermore, it can be utilized as a tool for discovering new factors that rescue pathogenic misfolding and aggregation, which could lead to the development of novel putative drugs.^32,33^ We anticipate that the findings reported in this study will facilitate the use of the MisP-GFP screen in future efforts to study disease-related protein misfolding and aggregation as well as the identification of new promising compounds in early-stage discovery programs against PMDs. It is expected that the MisP-GFP screen would be particularly efficient in identifying factors rescuing pathogenic misfolding and aggregation, which are selected from combinatorial biomolecular libraries following biosynthetic production in the same host as we have shown previously.^32,33^ For small-molecule libraries, hit identification would be limited to drugs which could cross bacterial membranes and gain access to the E. coli cytoplasm and also would not be metabolized or efficiently effluxed by E. coli cells.

## Methods

### Plasmid Construction

All enzymes for DNA cloning used in this study were purchased from New England Biolabs. Recombinant plasmids and PCR products/agarose extracted DNA were purified using the NucleoSpin Plasmid and the NucleoSpin Gel and PCR Clean-up kits, respectively, both from Macherey-Nagel. The pET28-Aβ42-GFP vector was a kind gift from Prof. M. H. Hecht (Princeton University). For the construction of pET28-T-p53C-GFP, the M133L, V203A, N239Y, and N268D mutations were incorporated into the p53C-encoding gene by overlap PCR using the pET28-p53Cwt-GFP^33^ as a template and primers p53for, p53rev, p53M133Lfor, p53M133Lrev, p53 V203Afor, p53 V203Arev, p53N239Yfor, p53N239Yrev, N268Dfor, and N268Drev (Supporting Information Table S3). The PCR product was then digested with NdeI and *Bam*HI and ligated into the similarly digested vector pETAβ42-GFP.^25^ To generate the constructs pET28-p53C­(V143A)-GFP, pET28-p53C­(Y220C)-GFP, and pET28-p53C­(F270L)-GFP, the p53C-encoding gene was mutated by overlap PCR using as a template pET28-p53Cwt-GFP and primers p53for, p53rev, p53Y220Cfor, p53Y220Crev, F270Lfor, F270Lrev, V143Afor, and V143Arev as appropriate. Then, the PCR product was digested with NdeI and *Bam*HI and inserted into pETAβ42-GFP, which had been similarly digested. For the construction of pET28-T-p53C­(V143A)-GFP and pETT-p53C­(Y220C)-GFP, the same approach was followed with the only variation being the use of pETT-p53C-GFP as the initial template instead of pET28-p53Cwt-GFP. In the case of pETT-p53C­(F270L)-GFP, a distinct set of primers, T-F270Lfor and T-F270Lrev, was also required for the mutagenesis process. For the construction of pASK75-T-p53C-GFP and pASK75-T-p53C­(Y220C)-GFP, the T-p53C-GFP and T-p53C­(Y220C)-GFP genes were amplified by PCR from the respective pET28 vectors using primers p53for and GFPrev and ligated into pASK75 using the restriction sites XbaI-*Hin*dIII. For the construction of pTrc-T-p53C-GFP, pTrc-T-p53C­(Y220C)-GFP, pBAD30-T-p53C-GFP, pBAD30-T-p53C­(Y220C)-GFP, pBAD18-T-p53C-GFP, and pBAD18-T-p53C­(Y220C)-GFP, the T-p53C-GFP and T-p53C­(Y220C)-GFP genes were digested from the respective pASK75 vectors using XbaI and *Hin*dIII and ligated into pTrc99a, pBAD30, or pBAD18 that were similarly digested. To generate the constructs pET28-T-p53C–BFP, pET28-T-p53C­(Y220C)–BFP, pET28-T-p53C-RFP, and pET28-T-p53C­(Y220C)-RFP, the BFP and RFP genes were amplified by PCR from pBADCstA-BFP and pSTC1 ^53^ (kind gift from Prof. Alfonso Jaramillo) using primers BFP­(*Bam*HI)­for/BFP­(XhoI)­rev and RFP­(*Bam*HI)­for/RFP­(XhoI)­rev, respectively. Then, the PCR products were digested with *Bam*HI and XhoI and inserted into similarly digested pET28-T-p53C-GFP and pET28-T-p53C­(Y220C)-GFP accordingly. To generate the constructs pET28-T-p53C-sfGFP and pET28-T-p53C­(Y220C)-sfGFP, the sfGFP gene was amplified by PCR pSTC1 and using primers sfGFP­(*Bam*HI)­for and sfGFP­(XhoI)­rev, the PCR product was then digested with *Bam*HI and XhoI and inserted into similarly digested pET28-T-p53C-RFP and pET28-T-p53C­(Y220C)-RFP. To generate the constructs pET28-T-p53C-GFPmut2 and pET28-T-p53C­(Y220C)-GFPmut2, the T-p53C and T-p53C­(Y220C) genes were digested with XbaI and *Pst*I from the respective pET-GFP vectors and ligated into similarly digested pET-BR2-GFP.^59^ For the construction of the pET28-TP53-GFP­(no linker) vectors, the *TP53* genes were amplified by PCR from the respective pET28-Tp53-GFP vectors using primers p53for and p53-GFP­(no linker)­rev, and the GFP gene was amplified by PCR using primers p53-GFP­(no linker)­for and GFP­(BsrGI)­rev. The two PCR products were then seamlessly ligated using overlap PCR and primers p53for and GFP­(BsrGI)­rev. Then, the final PCR products were digested using NdeI and BsrGI and ligated into the similarly digested pET28-Aβ42-GFP vector. To construct the pET28-TP53-GFP­((G_4_S)_2_^F^) vectors, the Tp53 and GFP genes were amplified from the respective pET28-Tp53-GFP vectors by PCR using primer pairs p53for/p53­(G4S)­2­(freq)­(*Bam*HI)­rev and GFP­(*Bam*HI)­for/GFP­(BsrGI)­rev. The PCR products were then digested with NdeI-*Bam*HI (for TP53) or *Bam*HI-BsrGI (for GFP) before three-way ligation into the NdeI/BsrGI digested pET28-Aβ42-GFP vector. The pET28-TP53-GFP­((G_4_S)_2_^R^) vectors were constructed in a similar manner with the only difference being the use of the p53­(G4S)­2­(rare)­(*Bam*HI)­rev primer in place of p53­(G4S)­2­(freq)­(*Bam*HI)­rev. Similarly, the pET28-TP53-GFP­(rigid linker) vectors were constructed by PCR extension of the Tp53 and GFP genes using the respective pET28-T-p53-GFP vectors as a template and primer pairs p53for/p53­(EAAAK)­3­(NotI)­rev and p53­(EAAAK)­3­(NotI)­for/GFP­(BsrGI)­rev and the PCR products were digested with NdeI-NotI (for TP53) or NotI-BsrGI (for GFP) before three-way ligation into the NdeI/BsrGI-digested pET28-Aβ42-GFP vector. In order to generate the insertional p53-GFP fusions, two sequential overlap PCRs were performed as such: (i) The N-terminus of GFP was amplified using pET28-T-p53C-GFP as a template and primers pairs GFP­(NdeI)­for/GFP-Tp53(157)­rev for the GFPi­(157/158) insertional fusion or GFP­(NdeI)­for/GFP-Tp53(172)­rev for the GFPi­(172/173) insertional fusion, (ii) the Tp53 genes were amplified using the appropriate pET28-T-p53-GFP as a template and primers pairs GFP-Tp53(157)­for/GFP-Tp53(158)­rev for GFPi­(157/158) or GFP-Tp53(172)­for/GFP-Tp53(173)­rev for GFPi­(172/173), (iii) PCR products from steps (i) and (ii) were ligated seamlessly using overlap PCR and primers GFP­(NdeI)­for/GFP-Tp53(158)­rev for GFPi­(157/158) or GFP­(NdeI)­for/GFP-Tp53(173)­rev for GFPi­(172/173), (iv) the C-terminus of GFP was amplified using pET28-T-p53C-GFP as a template and primers pairs GFP-Tp53(158)­for/GFP­(*Kpn*I)­rev for GFPi­(157/158) or GFP-Tp53(173)­for/GFP­(*Kpn*I)­rev for GFPi­(172/173), and finally (v) PCR products from steps (iii) and (iv) were ligated seamlessly using overlap PCR and primers GFP­(NdeI)­for/GFP­(*Kpn*I)­rev for both the GFPi­(157/158) and the GFPi­(172/173) insertional fusions. The PCR products from step (v) were then digested with NdeI-*Kpn*I and inserted into a similarly digested pET28-Aβ42-GFP vector. Plasmid construction information is summarized in Supporting Information Table S4.

### Protein Production in Liquid Cultures

E. coli BL21­(DE3) (F^–^ *ompT hsdS*_*B*_ (r_B_^–^, m_B_^–^) *gal dcm* (DE3)), Tuner­(DE3) (F^–^ *ompT hsdS*_B_ (r_B_^–^ m_B_^–^) *gal dcm lacY1*(DE3), or Origami 2­(DE3) (Δ*(ara-leu)­7697* Δ*lacX74* Δ*phoA Pvu*II *phoR araD139 ahpC galE galK rpsL* F′[*lac* ^*+*^ *lacI* ^*q*^ *pro*] (DE3) *gor522*:Tn*10 trxB* (Str^R^, Tet^R^) cells were freshly transformed with the appropriate expression vector, and single bacterial colonies were used to inoculate overnight liquid LB cultures containing the appropriate antibiotics for plasmid maintenance (100 μg/mL ampicillin, 50 μg/mL kanamycin, or 100 μg/mL streptomycin (Sigma)) at 37 °C. These cultures were used with a 1:100 dilution to inoculate 5 mL fresh cultures in 25 mm × 150 mm culture tubes with the LB medium (5 g tryptone powder, 2.5 g yeast extract, and 5 g NaCl per L) containing the relevant antibiotic and grown at 37 °C to an OD600 of ∼0.4 with shaking, at which point protein production was initiated by the addition of the appropriate inducer (IPTG for pET28- and pTrc99a-based vectors, l­(+)-arabinose for pBAD-based vectors, and anhydrotetracycline for pASK75-based vectors), as indicated in the manuscript text for each experiment. Recombinant protein production was performed at 37 °C for 2 h, 30 °C for 5 h, or 25 °C for 16 h, unless otherwise stated. After protein overexpression, bacterial cells corresponding to 1 mL culture with OD600 = 1 were harvested by centrifugation at 6000*g* for 2 min.

### Bacterial Cell Fluorescence

Bacterial fluorescence was measured either using a TECAN Safire II-Basic plate reader (Tecan, Austria), in which case cells were first harvested by centrifugation, resuspended in 100 μL phosphate-buffered saline (PBS), transferred to a 96-well FLUOTRAC 200 plate (Greiner Bio One International, Austria), and measured at 510 nm after excitation at 488 nm, or using a CyFlow ML flow cytometer (Partec), in which case cells were diluted in PBS to a final concentration of 10^5^–10^6^ cells/ml, and their fluorescence was recorded at 530/30 nm after GFP excitation at 488 nm and analyzed statistically using FlowJo vX.0.7.

### Western Blot Analyses

Cells corresponding to 1 mL of culture with OD600 = 1 were resuspended in 100 μL of PBS and lysed by brief sonication cycles on ice. Then, the resulting lysates (i.e., total fraction) were clarified by centrifugation at 13,000 rpm for 25 min, resulting in the soluble fraction and the insoluble cell pellet. Samples were boiled for 10 min and analyzed by SDS-PAGE on 10% gels. Then, proteins were transferred to polyvinylidene fluoride (PVDF) membranes (Merck, Germany) using a semidry blotter (Thermo Fisher, USA) for 1 h at 12 V. The membranes were subsequently blocked with 5% nonfat dry milk in Tris-buffered saline containing 0.1% Tween-20 (TBST), incubated with a mouse anti-GFP primary antibody (Clontech, USA) at a 1:20,000 dilution, and then incubated with a horseradish peroxidase (HRP)-conjugated goat antimouse secondary antibody (BioRad) at a 1:4000 dilution. All steps were performed for 1 h at room temperature, and between each step, the membranes were thoroughly washed with TBST. Finally, proteins were visualized using the ChemiDoc-It^2^ Imaging System (UVP, UK).

### Statistical Analyses

All graphs were prepared using Prism (GraphPad Software Inc., USA), and mean values of one experiment performed in triplicate with standard deviations are presented, unless otherwise stated.

## Supplementary Material



## Notes

Thermodynamic stability of the p53 core domain variants; application of the MisP-GFP genetic screen to monitor misfolding and aggregation for other MisPs-the case of Αβ; plasmids used in this study; specifications of linkers used in this study; PCR primers used in this study; and synopsis of the construction process of the plasmids used in this study (PDF)
