# Supplementary material for: Optimization of a High-Throughput Screen for Monitoring Disease-Associated Protein Misfolding and Aggregation in Bacteria
Source: ACS Synth Biol. 2025 May 12;14(6):2283–93. doi: 10.1021/acssynbio.5c00166 (PMC12186678; doi:10.1021/acssynbio.5c00166)
Supplement: Supplementary file 1 [file sb5c00166_si_001.pdf]

Supplementary information

for the article

Optimization of a high-throughput genetic screen for monitoring protein misfolding and aggregation in bacteria

Dafni C. Delivoria[1], Eleni Konia[2,3], Ilias Matis[1] and Georgios Skretas[1,2*]

[1]*Institute of Chemical Biology, National Hellenic Research Foundation, 11635 Athens, Greece*

[2]*Institute for Bio-innovation, Biomedical Sciences Research Center "Alexander Fleming", 16672 Vari, Greece*

[3]*Department of Chemistry, University of Crete, Iraklio, Crete 70013, Greece*

* To whom correspondence should be addressed

Georgios Skretas

Institute for Bio-innovation

Biomedical Sciences Research Center "Alexander Fleming"

34 Fleming st.

16672 Vari

Greece

Tel: +302109656310 (ext. 182)

skretas@fleming.gr

Supplementary figures

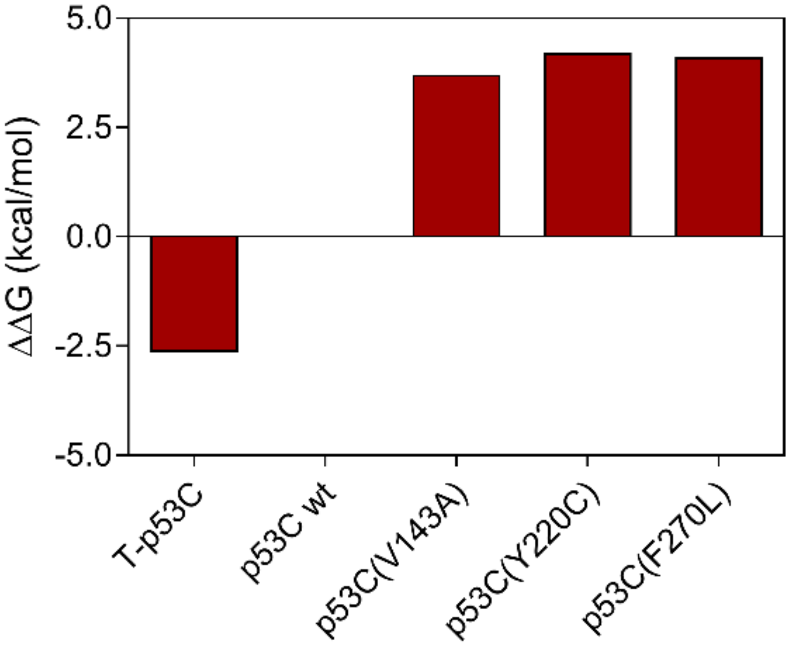

Supplementary Figure 1. Thermodynamic stability of the p53 core domain variants. ΔΔG represents the change in the free energy of urea-induced unfolding caused by mutations in wild-type p53 (ΔΔG= ΔGwt – ΔGmut) as determined by Fersht and co-workers [1, 2].

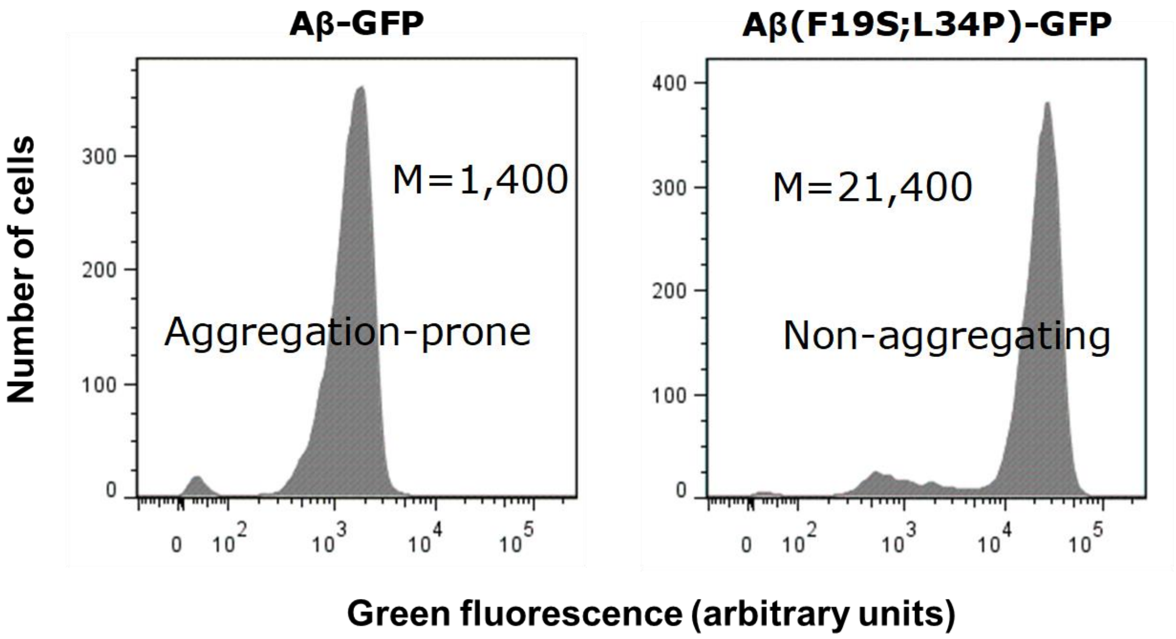

Supplementary Figure 2. Application of the MisP-GFP genetic screen to monitor misfolding and aggregation for other MisPs – the case of Aβ. Comparison of fluorescence of *E. coli* BL21(DE3) cells over-expressing Aβ42-GFP fusions containing wild-type Aβ42 (Aβ) and the less aggregation-prone variant F19S/L34P using the pET28-Aβ42-GFP vector following induction of protein overexpression by the addition of 0.1 mM IPTG for 2 h at 37 ^{o}C, as measured by flow cytometry.

Supplementary tables

Supplementary Table 1. Plasmids used in this study.

**Plasmid**	**Protein variants expressed**	**Promoter**	**Origin of replication**	**RP**	**Source**
p53					
pET28-p53-GFP	wt, Y220C	T7	pBR322	GFP+	Matis et al. [3]
	V143A, F270L, T-p53C, T-p53C (Y220C), T-p53C (V143A), T-p53C(F270L)				This work
pASK75- p53-GFP	T-p53C, T-p53C(Y220C)	Tet	pBR322	GFP+	This work
pTrc99A- p53-GFP	T-p53C, T-p53C(Y220C)	trc	pBR322	GFP+	This work
pBAD30- p53-GFP	T-p53C, T-p53C(Y220C)	araBAD	pACYC	GFP+	This work
pBAD18- p53-GFP	T-p53C, T-p53C(Y220C)	araBAD	pBR322	GFP+	This work
pET28- p53-GFPmut2	T-p53C, T-p53C(Y220C)	T7	pBR322	GFPmut2	This work
pET28- p53-sfGFP	T-p53C, T-p53C(Y220C)	T7	pBR322	sfGFP	This work
pET28- p53-BFP	T-p53C, T-p53C(Y220C)	T7	pBR322	BFP	This work
pET28- p53-RFP	T-p53C, T-p53C(Y220C)	T7	pBR322	RFP	This work
pET28-p53-GFP(no linker)	T-p53C, T-p53C(Y220C)	T7	pBR322	GFP+	This work
pET28-p53-GFP(Gly$_4$/Ser)$_2^R$	T-p53C, T-p53C(Y220C)	T7	pBR322	GFP+	This work
pET28-p53-GFP(Gly$_4$/Ser)$_2^F$	T-p53C, T-p53C(Y220C)	T7	pBR322	GFP+	This work
pET28-p53-GFP(rigid linker)	T-p53C, T-p53C(Y220C)	T7	pBR322	GFP+	This work
pET28-p53-GFPi(157/158)	T-p53C, T-p53C(Y220C)	T7	pBR322	GFP+	This work
pET28-p53-GFPi(172/173)	T-p53C, T-p53C(Y220C)	T7	pBR322	GFP+	This work
Aβ					

pET28- Aβ42-GFP	Aβ42, Aβ42(F19S; L34P)	T7	pBR322	GFP+	Matis et al. [3]
SOD1					
pET28- SOD1-GFP	wt, A4V, G37R, G85R, G93A	T7	pBR322	GFP+	Matis et al. [3]
pASK75- SOD1-GFP	wt, A4V, G37R, G85R, G93A	T7	pBR322	GFP+	Matis et al. [3]

Supplementary Table 2. Specifications of linkers used in this study.

**Linker name**	**Characteristics**	**DNA sequence**	**Peptide sequence**	**Peptide size**
Waldo(ext)	Flexible linker, rich in small and polar amino acids such as Gly, Ala, and Ser, promoting MisP-RP solubility	CTG CAG GGA TCC GCT GGC TCC GCT GCT GGT TCT GGC GAA TTC	LQGSA GSAAGS GEF	14 aa
$(Gly_4/Ser)_2^R$	Flexible linker, rich in Gly and Ser from rare codons, decelerating translation and promoting MisP folding	GGG GGA GGG GGA TCA GGG GGA GGG GGA TCC	GGGGS GGGGS	10 aa
$(Gly_4/Ser)_2^F$	Flexible linker, rich in Gly and Ser from frequent codons for accelerated translation, promoting MisP misfolding	GGC GGT GGC GGT TCG GGC GGT GGC GGA TCC	GGGGS GGGGS	10 aa
$(EAAAK)_3$	Rigid linker with helical structure to hinder MisP-RP interaction	GAA GCG GCG GCC AAA GAA GCG GCC GCG AAA GAA GCC GCC GCG AAA	EAAAK EAAAK EAAAK	15 aa

Supplementary Table 3. PCR primers used in this study.

Name	Primer sequence (5'-3')	Use
p53for	AAAAAATCTAGAAGGAGGAAACGCATATGTCATCTTCTGTCCCTTCCCAG	Forward primer for the construction of p53 constructs containing a XbaI (double underlined) and a NdeI site (underlined)
p53rev	AAAAAAGGATCCCTGCAGGGTGTTGTTGGACAGTGCTCG	Reverse primer for the construction of the p53 protein fusion with GFP containing a BamHI site (underlined)
p53M133Lfor	CCTGCCCTCAACAAGCTGTTTTGCCAACTGGCC	Forward point mutagenesis primer for the M133L mutation in the sequence of p53C
p53M133Lrev	GGCCAGTTGGCAAAACAGCTTGTTGAGGGCAGG	Reverse point mutagenesis primer for the M133L mutation in the sequence of p53C
p53V203Afor	GGAAATTTGCGTGCGGGAGTATTTGGATGAC	Forward point mutagenesis primer for the V203A mutation in the sequence of p53C
p53V203Arev	GTCATCCAAATACTCCCGCACGCAAATTTCC	Reverse point mutagenesis primer for the V203A mutation in the sequence of p53C
p53N239Yfor	CAACTACATGTGTTACAGTTCCTGCATGGGC	Forward point mutagenesis primer for the N239Y mutation in the sequence of p53C
p53N239Yrev	GCCCATGCAGGAACTGTAACACATGTAGTTG	Reverse point mutagenesis primer for the N239Y mutation in the sequence of p53C
N268Dfor	GTAATCTACTGGGACGGGACAGCTTTGAGGTGCGTG	Forward point mutagenesis primer for the N268D mutation in the sequence of p53C
N268Drev	CACGCACCTCAAAGCTGTCCCGTCCCAGTAGATTAC	Reverse point mutagenesis primer for the N268D mutation in the sequence of p53C

p53Y220Cfor	AGTGTGGTGGTGCCCTGTGAGCCGCCTGAGGTTG	Forward point mutagenesis primer for the Y220C mutation in the sequence of p53C
p53Y220Crev	CAACCTCAGGCGGCTCACAGGGCACCACCACACT	Reverse point mutagenesis primer for the Y220C mutation in the sequence of p53C
F270Lfor	CTGGGACGGAACAGCTTAGAGGTGCGTGTTTGTG	Forward point mutagenesis primer for the F270L mutation in the sequence of p53C
F270Lrev	CACAAACACGCACCTCTAAGCTGTTCCGTCCCAG	Reverse point mutagenesis primer for the F270L mutation in the sequence of p53C
V143Afor	GCCAAGACCTGCCCTGCGCAGCTGTGGGTTGATTC	Forward point mutagenesis primer for the V143A mutation in the sequence of p53C
V143Arev	GAATCAACCCACAGCTGCGCAGGGCAGGTCTTGGC	Reverse point mutagenesis primer for the V143A mutation in the sequence of p53C
T-F270Lfor	CTGGGACGGGACAGCTTAGAGGTGCGTGTTTGTG	Forward point mutagenesis primer for the F270L mutation in the sequence of T-p53C
T-F270Lrev	CACAAACACGCACCTCTAAGCTGTCCCGTCCCAG	Reverse point mutagenesis primer for the F270L mutation in the sequence of T-p53C
GFPrev	AAAAAAAAGCTTCTCGAGTTAGTGGTGGTGGTGGTGGTGTTTGTAGAGTTCATCCATGCC	Reverse primer for the construction of GFP fused protein constructs containing a HindIII site (underlined) and a XhoI site (double underlined)
BFP(BamHI) for	AAAAAGGATCCGGTTCTGGTTCTATGAGCGAAGAACTGATCAAAG	Forward primer for the introduction of BFP downstream of p53 variants containing a BamHI site (underlined)
BFP(XhoI)rev	AAAAACTCGAGAAGCTTTTAGTTCAGTTTGTGACCCAGTTTAG	Reverse primer for the introduction of BFP downstream of p53 variants

		containing a HindIII (underlined) and a XhoI site (double underlined)
RFP (BamHI) for	AAAAAGGATCCGGTTCTGGTTCTGCTTCCTCCGAAGACGTTATC	Forward primer for the introduction of RFP downstream of p53 variants containing a BamHI site (underlined)
RFP(XhoI)rev	TTTTTCTCGAGTTAAGCACCGGTGGAGTGACGAC	Reverse primer for the introduction of RFP downstream of p53 variants containing a XhoI site (underlined)
sfGFP(BamHI)for	AAAAAGGATCCGGTTCTGGTTCTCGTAAAGGCGAAGAGCTGTTC	Forward primer for the introduction of sfGFP downstream of p53 variants containing a BamHI site (underlined)
sfGFP(XhoI)rev	TTTTTCTCGAGAAGCTTTTATTTGTACAGTTCATCCATAC	Reverse primer for the introduction of sfGFP downstream of p53 variants containing a HindIII (underlined) and a XhoI site (double underlined)
p53(NcoI)For	AAAAACCATGGTTTCATCTTCTGTCCCTTCCCAG	Forward primer for the introduction of p53 into the pCDF-1b vector containing a NcoI site (underlined)
Tp53(ΔNcoI)for	CACCCGCGTCCGCGCAATGGCCATCTACAAG	Forward point mutagenesis primer for the elimination of the NcoI site in the sequence of T-p53C
Tp53(ΔNcoI)rev	CTTGTAGATGGCCATTGCGCGGACGCGGGTG	Reverse point mutagenesis primer for the elimination of the NcoI site in the sequence of T-p53C
p53$(G_4S)_2$(rare)(BamHI)rev	AAAAAGGATCCCCCTCCCCCTGATCCCCCTCCCCCGGTGTTGTTGGACAGTGCTC	Reverse primer for the introduction of the $(G_4S)_2^R$ linker between TP53 and GFP and containing a BamHI site (underlined)
p53$(G_4S)_2$(freq)(BamHI)rev	AAAAAGGATCCGCCACCGCCCGAACCGCCACCGCCGGTGTTGTTGGACAGTGCTC	Reverse primer for the introduction of the $(G_4S)_2^F$ linker between TP53 and GFP and containing a BamHI site (underlined)

GFP(BamHI)for	AAAAAGGATCCAGCAAAGGAGAAGAACTTTTC	Forward primer for the introduction of the $(G_4S)_2$ linkers between TP53 and GFP and containing a BamHI site (underlined)
GFP(BsrGI)rev	AAAAATGTACATAACCTTCGGGCATGGCAC	Reverse primer for the introduction of TP53-GFP fusions with different linkers into pET28-GFP and containing a BsrGI site (underlined)
p53-GFP(no linker)for	CACTGTCCAACAACACCAGCAAAGGAGAAGAAC	Forward primer for seamless ligation of TP53 genes with GFP
p53-GFP(no linker)rev	GTTCTTCTCCTTTGCTGGTGTTGTTGGACAGTG	Reverse primer for seamless ligation of TP53 genes with GFP
p53$(EAAAK)_3$(NotI)rev	AAAAAGCGGCCGCTTCTTTGGCCGCCGCTTCGGTGTTGTTGGACAGTGCTC	Reverse primer for the introduction of the $(EAAAK)_3$ linker between TP53 and GFP and containing a NotI site (underlined)
p53$(EAAAK)_3$(NotI)for	AAAAAGCGGCCGCGAAAGAAGCCGCCGCGAAAAGCAAAGGAGAAGAACTTTTC	Forward primer for the introduction of the $(EAAAK)_3$ linker between TP53 and GFP and containing a NotI site (underlined)
GFP(NdeI)for	AAAAACATATGAGCAAAGGAGAAGAACTTTTC	Forward primer for the introduction of GFPi-TP53 fusions into pET28 and containing a NdeI site (underlined)
GFP(KpnI)rev	AAAAAGGTACCTTATTTGTAGAGCTCATCCATG	Reverse primer for the introduction of GFPi-TP53 fusions into pET28 and containing a KpnI site (underlined)
GFP-Tp53(157)for	GTATACATCACGGCAGACAAACAATCATCTTCTGTCCCTTCCCAG	Forward primer for the introduction of TP53 genes after Gln157 of GFP
GFP-Tp53(157)rev	CTGGGAAGGGACAGAAGATGATTGTTTGTCTGCCGTGATGTATAC	Reverse primer for the introduction of TP53 genes after Gln157 of GFP

GFP-Tp53(158)for	GAGCACTGTCCAACAACACCAAGAATGGAATCAAAGCTAAC	Forward primer for the introduction of TP53 genes before Lys158 of GFP
GFP-Tp53(158)rev	GTTAGCTTTGATTCCATTCTTGGTGTTGTTGGACAGTGCTC	Reverse primer for the introduction of TP53 genes before Lys158 of GFP
GFP-Tp53(172)for	CAAAATTCGCCACAACATTGAATCATCTTCTGTCCCTTCCCAG	Forward primer for the introduction of TP53 genes after Glu172 of GFP
GFP-Tp53(172)rev	CTGGGAAGGGACAGAAGATGATTCAATGTTGTGGCGAATTTTG	Reverse primer for the introduction of TP53 genes after Glu172 of GFP
GFP-Tp53(173)for	GAGCACTGTCCAACAACACCGATGGTTCCGTTCAACTAGC	Forward primer for the introduction of TP53 genes before Asp173 of GFP
GFP-Tp53(173)rev	GCTAGTTGAACGGAACCATCGGTGTTGTTGGACAGTGCTC	Reverse primer for the introduction of TP53 genes before Asp173 of GFP

Supplementary Table 4. Synopsis of the construction process of the plasmids used in this study.

Construct Name	Gene source	PCR primers	Enzymes used	Destination Vector
pET28-T-p53C-GFP	pET28-p53Cwt-GFP	p53for, p53rev, p53M133Lfor, p53M133Lrev, p53V203Afor, p53V203Arev, p53N239Yfor, p53N239Yrev, N268Dfor and N268Drev	NdeI, BamHI	pETAβ42-GFP
pET28-p53C(V143A)-GFP	pET28-p53Cwt-GFP	p53for, p53rev, V143Afor and V143Arev	NdeI, BamHI	pETAβ42-GFP
pET28-p53C(Y220C)-GFP	pET28-p53Cwt-GFP	p53for, p53rev, p53Y220Cfor, p53Y220Crev,	NdeI, BamHI	pETAβ42-GFP
pET28-p53C(F270L)-GFP	pET28-p53Cwt-GFP	p53for, p53rev, F270Lfor, F270Lrev	NdeI, BamHI	pETAβ42-GFP
pET28-T-p53C(V143A)-GFP	pET28-T-p53C-GFP	p53for, p53rev, V143Afor and V143Arev	NdeI, BamHI	pETAβ42-GFP
pET28-T-p53C(Y220C)-GFP	pET28-T-p53C-GFP	p53for, p53rev, p53Y220Cfor, p53Y220Crev,	NdeI, BamHI	pETAβ42-GFP
pET28-T-p53C(F270L)-GFP	pET28-T-p53C-GFP	p53for, p53rev, T-F270Lfor and T-F270Lrev	NdeI, BamHI	pETAβ42-GFP
pASK75-TP53-GFP	pET28-TP53-GFP	p53for and GFPrev	XbaI, HindIII	pASK75
pTrc-TP53-GFP,	pASK75 - TP53-GFP	-	XbaI, HindIII	pTrc99a
pBAD30-TP53-GFP,	pASK75 - TP53-GFP	-	XbaI, HindIII	pBAD30
pBAD18-TP53-GFP	pASK75 - TP53-GFP	-	XbaI, HindIII	pBAD18
pET28-TP53-BFP,	pBADCstA-BFP	BFP(BamHI)for/BFP(XhoI)rev	BamHI, XhoI	pET28-TP53-EGFP

pET28-TP53-RFP	T_YES_Isaacs_1_pSTC1	RFP(BamHI)for/RFP(XhoI)rev	BamHI, XhoI	pET28-TP53-EGFP
pET28-TP53-sfGFP	T_YES_Isaacs_1_pSTC1	sfGFP(BamHI)for and sfGFP(XhoI)rev	BamHI, XhoI	pET28- TP53-RFP
pET28-TP53-GFPmut2	pET28-TP53-EGFP	-	XbaI, PstI	pET-BR2-GFP
pET28-TP53-GFP(no linker)	pET28-TP53-GFP	p53for and p53-GFP(no linker)rev, p53-GFP(no linker)for and GFP(BsrGI)rev, p53for and GFP(BsrGI)rev	NdeI, BsrGI	pET28-Aβ42-GFP
pET28-TP53-GFP((G_4S)$_2^F$)	pET28-TP53-GFP	p53for/p53(G4S)2(freq)(BamHI)rev and GFP(BamHI)for/ GFP(BsrGI)rev	NdeI, BamHI, BsrGI	pET28-Aβ42-GFP
pET28-TP53-GFP((G_4S)$_2^R$)	pET28-TP53-GFP	p53for/p53(G4S)2(rare)(BamHI)rev and GFP(BamHI)for/ GFP(BsrGI)rev	NdeI, BamHI, BsrGI	pET28-Aβ42-GFP
pET28-TP53-GFP(rigid linker)	pET28-TP53-GFP	p53for/p53(EAAAK)3(NotI)rev and p53(EAAAK)3(NotI)for/ GFP(BsrGI)rev	NdeI, NotI, BsrGI	pET28-Aβ42-GFP
GFPi(157/158)	pET28-T-p53C-GFP	GFP(NdeI)for/GFP-Tp53(157)rev, GFP-Tp53(157)for/GFP-Tp53(158)rev, GFP(NdeI)for/GFP-Tp53(158)rev, GFP-Tp53(158)for/ GFP(KpnI)rev, GFP(NdeI)for/GFP(KpnI)rev	NdeI, KpnI	pET28-Aβ42-GFP
GFPi(172/173)	pET28-T-p53C-GFP	EGFP(NdeI)for/GFP-Tp53(172)rev, GFP-Tp53(172)for/GFP-Tp53(173)rev, EGFP(NdeI)for/GFP-Tp53(173)rev, GFP-Tp53(173)for/GFP(KpnI)rev, GFP(NdeI)for/GFP(KpnI)rev	NdeI, KpnI	pET28-Aβ42-GFP

Supplementary references

1. Joerger, A. C.; Ang, H. C.; Fersht, A. R., Structural basis for understanding oncogenic p53 mutations and designing rescue drugs. *Proc Natl Acad Sci U S A* **2006,** *103* (41), 15056-61.

2. Bullock, A. N.; Henckel, J.; Fersht, A. R., Quantitative analysis of residual folding and DNA binding in mutant p53 core domain: Definition of mutant states for rescue in cancer therapy. *Oncogene* **2000,** *19* (10), 1245-56.

3. Matis, I.; Delivoria, D. C.; Mavroidi, B.; Papaevgeniou, N.; Panoutsou, S.; Bellou, S.; Papavasileiou, K. D.; Linardaki, Z. I.; Stavropoulou, A. V.; Vekrellis, K.; Boukos, N.; Kolisis, F. N.; Gonos, E. S.; Margarity, M.; Papadopoulos, M. G.; Efthimiopoulos, S.; Pelecanou, M.; Chondrogianni, N.; Skretas, G., An integrated bacterial system for the discovery of chemical rescuers of disease-associated protein misfolding. *Nat Biomed Eng* **2017,** *1* (10), 838-852.
